# Profil épidémiologique et clinique des enfants atteints de la maladie à coronavirus (COVID-19) au Centre de Traitement des Epidémies et Prévention des Infections (CTEPI) du CHU de Donka à Conakry

**DOI:** 10.11604/pamj.2020.37.363.26211

**Published:** 2020-12-21

**Authors:** Emmanuel Camara, Ibrahima Koolo Barry, Fatoumata Binta Diallo, Mohamed Lamine Diallo, Mamadou Moustapha Diop, Mahamoud Sama Cherif, Ibrahima Sory Diallo, Moustapha Kouyaté, Mmah Aminata Bangoura, Aissata Barry, Mamadou Ciré Barry, Ipolithe Honde Ngadande, Oumar Kaba, Ouo Ouo Kolié, Diarra Doura Camara, Saliou Bella Diallo, Hasmiou Dia

**Affiliations:** 1 Service de Pédiatrie, Hôpital National Donka, Conakry, Guinée,; 2 Institut de Nutrition et de Santé de l´Enfant, Conakry, Guinée,; 3 Service des Urgences Pédiatriques, Hôpital National Donka, Conakry, Conakry Guinée,; 4 Service de Pédiatrie, Hôpital National Ignace Deen, Conakry, Guinée

**Keywords:** COVID-19, enfants, épidémiologique, CTPI, Donka, COVID-19, children, epidemiological, CTEPI, Donka

## Abstract

La COVID-19 est due au virus SARS-CoV-2 génétiquement semblable au virus du syndrome respiratoire aigu sévère (SARS). En pédiatrie, les formes cliniques ont un caractère bénin. En Guinée, suite à la pandémie du COVID-19 dont l´épicentre était Conakry, des cas pédiatriques ont été observés au CTEPI de Donka. L´objectif de cette étude était de déterminer leur profil épidémiologique. Etude transversale de type descriptif de 04 mois chez les enfants de 0 à 16 ans admis au CTEPI Donka. Sur 7308 patients provenant principalement des 5 communes de Conakry hospitalisés au CTEPI, 189 étaient âgés de 0 à 16 ans, soit 2, 59%. La tranche de 0-4 ans était plus représentée (38,62%) avec un sex-ratio (F/M) de 1,52; 62,96% étaient des élèves, 70% des enfants résidaient à Conakry, 28,57% des mères étaient des marchandes et personnes contactes dans 39,68%; 37,57% des pères étaient des fonctionnaires, 2,65% des enfants avaient des antécédents de drépanocytose et 1,59% de rhinite allergique. Les cas asymptomatiques représentaient 52,38% et les cas confirmés 74, 6%; les symptômes étaient: fièvre, rhinorrhée, céphalées, toux, douleurs abdominales, éternuement, diarrhée, asthénie physique. La proportion des cas pédiatriques infectés au COVID-19 au CTEPI de Donka est faible. Les enfants de 5 ans et plus sont plus concernés et près de 50% était asymptomatique. Les symptômes dominants sont: fièvre, céphalées, rhinorrhée, toux, douleurs abdominales, éternuement, diarrhée, asthénie physique.

## Abstract (en)

COVID-19 is caused by SARS-CoV-2 virus, which is genetically similar to severe acute respiratory syndrome (SARS) virus. In pediatrics, it has a benign clinical course. Since the outbreak of COVID-19 pandemic in Guinea, whose epicenter was Conakry, pediatric cases have been reported at the CTEIP in Donka. The purpose of this study was to determine their epidemiological profile. We conducted a descriptive cross-sectional study of children aged 0-16 years admitted to the CTEIP, Donka, over a period of four months. Out of 7308 patients, coming predominantly from 5 communes of Conakry and hospitalized in the CTEPI, 189 were aged between 0 and 16 years (2 .59%). The majority of patients were within the age-group 0-4-years (38.62%) with a sex-ratio (F/M) of 1.52; 62.96% were students, 70% of children lived in Conakry, 28.57% of mothers were traders and contact persons (39.68%); 37.57% of fathers were civil servants, 2.65% of children had a history of sickle cell disease and 1.59% had allergic rhinitis. Asymptomatic patients accounted for 52.38% of cases and diagnosed patients were 74.6%. Symptoms included fever, rhinorrhea, headache, cough, abdominal pain, sneezing, diarrhea, physical asthenia. The incidence of COVID-19 among children hospitalized in the CTEIP of Donka is low. Children aged 5 years and older are more affected and nearly 50% are asymptomatic. Common symptoms are fever, headache, rhinorrhea, cough, abdominal pain, sneezing, diarrhea, physical asthenia.

## Introduction

La maladie à coronavirus 2019 (COVID-19) est une maladie, due à un nouveau virus qui a été nommé SARS-CoV-2 (11 février 2020). Ce virus est génétiquement semblable à ceux responsables du syndrome respiratoire aigu sévère (SARS) de 2003 et du syndrome respiratoire du Moyen-Orient [1]. En décembre 2019, les premiers cas de COVID-19 ont émergé dans la région de Wuhan, en Chine, où des personnes ont manifesté des symptômes de pneumonie sévère (p. ex., fièvre, toux, dyspnée, hémoptysie) [2]. La pandémie de la COVID-19 a été déclarée crise sanitaire mondiale par l´Organisation Mondiale de la Santé (OMS) fin janvier 2020 [3]. Des mesures exceptionnelles de confinement ont été prises pour contenir cette infection très contagieuse et parfois grave que les équipes soignantes combattent en première ligne. Selon les prévisions faites à partir des données reçues des pays touchés dès le début de la pandémie, 40% des cas développeront une maladie bénigne, 40% seront atteints d´affections de formes modérées, notamment la pneumonie, tandis que 15% des cas souffriront d´affections de formes graves, et les 5% restant seront inscrits au nombre des cas critiques de COVID-19.

En date du 05 juillet 2020, on comptait officiellement 11 125 245 cas et 528 204 décès à travers le monde [4]. De plus grandes séries d´études confirment le caractère bénin des formes cliniques de la maladie chez les enfants [5]. Quand les enfants font l´objet d´un dépistage ciblé en raison de leurs symptômes ou parce qu´ils ont été en contact avec des cas intrafamiliaux; la proportion de cas positifs par RT-PCR est la même que celle des adultes pour les enfants de 10 à 19 ans autour de 15% plus élevée pour les garçons que pour les filles [6]. Cette proportion est en revanche plus faible (6%), et non différente entre garçons et filles, pour les enfants de moins de 10 ans [7]. Les enfants présentent plus volontiers des formes ORL que des formes pulmonaires. Ils peuvent également être porteurs sains. Plusieurs études rapportent que les enfants, quelle que soit la forme clinique, peuvent garder du virus dans le nez et la gorge pour une période de 9 à 11 jours [8]. L´excrétion du virus dans les selles est fréquente, en l´absence de diarrhée, et peut durer jusqu´à 30 jours, sans que l´on sache si le virus est infectant ou non [8]. En Guinée, malgré les efforts consentis dans l´amélioration de la santé des populations en générale et celle de la population pédiatrique en particulier les cas de COVID-19 ont souvent été observés chez les enfants au CTEPI de Donka. Cette étude visait à déterminer le profil épidémiologique du COVID-19 chez les enfants de 0 à 16 ans hospitalisés dans ce centre.

## Méthodes

Il s´agissait d´une étude transversale de type descriptif effectuée au CTEPI de l´hôpital national Donka pendant une période de 04 mois allant du 1^er^ avril 2020 au 31 juillet 2020. L´hôpital national Donka (HND) est situé à Conakry (capitale) et constitue avec l´hôpital national Ignace Deen le CHU de la faculté des sciences et techniques de la santé (FSTS) de l´université Gamal Abdel Nasser de Conakry (UGANC). L´HND se situe au sommet de la pyramide sanitaire en Guinée, ce qui lui confère le titre d´hôpital de référence. Au sein de l´HND est créé un CTPI-COVID-19 pour la prise en charge (diagnostic PCR et traitement) des cas suspects ou avérés de COVID-19 référés par les différentes structures de santé de la région de Conakry et environnantes. Dans ces structures s´effectue le tri des cas suspects et la référence vers le CTPI de l´HND et cela sans distinction d´âge et de sexe.

Au cours de cette étude ont été inclus tous les enfants âgés de 0-16 ans (suspectés ou atteints de COVID-19) reçus en consultation et/ou hospitalisés au CTPI de Donka. Les critères d´admission des enfants étaient les suivants: les cas confirmés (enfants asymptomatiques ou symptomatiques dont la PCR au COVID-19 est positive); les cas suspects (enfants présentant soit une toux, ou un écoulement nasal, une difficulté respiratoire, une fièvre ≥38°C,….) mais dont le test PCR est négatif ou n´a pas été effectué; les cas contacts (enfants asymptomatiques avec une PCR négative au COVID-19 ayant été en contact avec un cas confirmé).

Le test PCR au COVID-19 était effectué à partir des prélèvements naso-pharyngés. Une fiche d´enquête structurée préétablie a permis de recueillir les données sociodémographiques des enfants, des parents ou des personnes qui assument leurs charges (Ages, sexe, provenance par commune/préfecture, profession des parents, personnes contactes); des données cliniques (antécédents pathologiques des enfants, motifs de consultation, cas cliniques retrouvés). Les données ont été saisies et traitées à l´aide du logiciel EPI info 7.2 puis présentées sous forme de tableaux à l´aide des logiciels Word et Excel du pack Office 2010. Les variables qualitatives ont été exprimées en pourcentage et les variables quantitatives en moyenne avec les extrêmes. L´anonymat et la confidentialité des informations recueillies ont été préservés.

## Résultats

**Figure 1:**
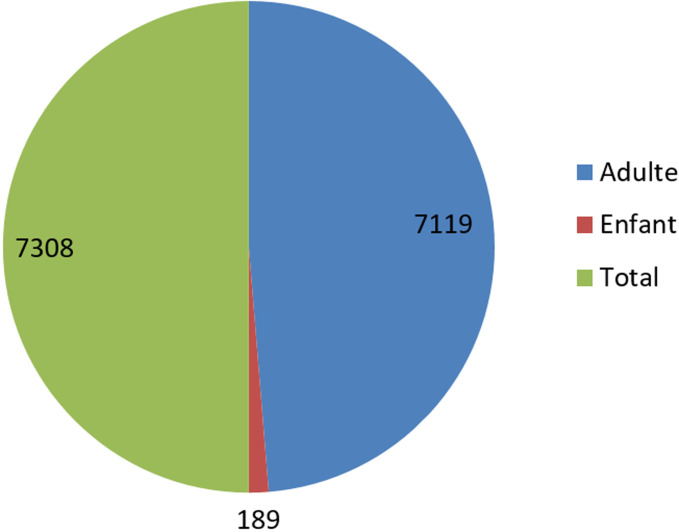
répartition des patients hospitalisés au CTEPI Donka du 1^er^ avril 2020 au 31 juillet 2020

Sur un total de 7308 patients hospitalisés au CTPI de l´HND, on a dénombré 189 enfants âgés de 0 à 16 ans (2.59%), ces enfants avaient en commun le fait d´avoir au moins eu l´un des proches hospitalisé pour COVID-19 au CTPI. La tranche d´âge 0-4 ans a été la plus représentée (38,62%) avec des extrémités de 0 jour et 16 ans et un âge médian de 7,19 ans. Une nette prédominance féminine a été relevée avec un sex-ratio de 1,52. La majorité des enfants résidaient à Conakry (70,37%) principalement dans les communes de Ratoma (42,33%) et de Matoto (28,04%). Près de 63% des enfants positifs au COVID-19 étaient des élèves, un 1/3 des mères (28,57%) étaient des marchandes et représentaient 40% des personnes contactes (Figure 1).

Les professions des pères étaient dominées par les fonctionnaires (37,57%). De faibles proportions d´enfants (2,65%) avaient comme antécédent connus la drépanocytose et de la rhinite allergique (1,59%). Les enfants asymptomatiques représentaient 52,38% de notre échantillon et les cas confirmés 74, 60%. Les principaux symptômes étaient la fièvre, la rhinorrhée, les céphalées, la toux, les douleurs abdominales, l´éternuement, la diarrhée, l´asthénie physique; la durée moyenne des symptômes de 6 (±2) jours. Le traitement était à base de l´hydroxychloroquine en raison de 10mg/kg en 3 prises par jour pendant 6 jours, l´azithromycine 20mg/kg en 1 prise par jour pendant 5 jours et le Zinc 20mg par jour pour les enfants de 6 mois et plus, 10mg par jour pour les moins de 6 mois pendant 10 jours. L´évolution avait été favorable pour l´ensemble des enfants avec aucun cas de décès. Les principales caractéristiques sociodémographiques et cliniques des enfants hospitalisés au CTEPI Donka du 1^er^ avril 2020 au 30 juin 2020 sont présentées respectivement aux Tableau 1et Tableau 2.

**Tableau 1:** caractéristiques sociodémographiques des enfants hospitalisés au CTEPI Donka 1^er^ avril 2020 au 31 juillet 2020

Variables	Effectifs (189)	Pourcentages
**Age (années), Médiane (7,19), extrême 0 et 16 ans**		
0-4 ans	73	38,62
5-10 ans	59	31,22
11- 16 ans	57	30,6
**Sexe**		
Masculin	75	39,68
Féminin	114	60,32
**Résidence**		
Ratoma	80	42,33
Matoto	53	28,04
Matam	12	6,35
Dixinn	12	6,35
Kaloum	7	3,70
Coya	13	6,88
Dubréka	8	4,23
Boké	3	1,59
Kindia	1	0,53
**Profession-des enfants**		
Non scolarisé	68	35,98
Elève	119	62,96
couturière	2	1,06
**Profession des mères**		
Marchande	54	28,57
Couturière	9	4,76
Coiffeuse	3	1,59
Fonctionnaire	50	26,46
Ménager	49	25,92
Etudiante	11	5,82
Enseignante	10	5,29
Coiffeuse	3	1,59
**profession des pères**		
Chauffeur	21	11,11
Fonctionnaire	71	37,57
Sans emploi	12	6,35
Marchand	52	27,51
Enseignant	7	3,70
Ouvrier	25	13,23
**Personnes contactes**		
Mère	75	39,68
Père	37	19,58
Père et mère	52	27,51
Frère	5	2,65

**Tableau 2:** caractéristiques cliniques des enfants hospitalisés au CTEPI Donka 1^er^ avril 2020 au 31 juillet 2020

Variables	Effectifs (189)	Pourcentages
**Antécédents**		
Drépanocytose	5	2,65
Rhinite allergique	3	1,59
Dermatite atopique	1	0,53
Néphroblastome	1	0,53
**Modalités d'admission**		
Cas confirmé	141	74,60
Cas suspect	18	9,52
Cas contact	30	15,87
Asymptomatique	99	52,38
symptomatique	90	47,62
**Motifs de consultation**		
Fièvre	41	21,69
Nez qui coule	37	19,58
Toux	25	13,23
Céphalées	31	16,40
Diarrhée	7	3,70
Vomissement	6	3,17
Dyspnée	4	2,12
Mal à la gorge	9	4,76
Otalgie	4	3,17
Rougeur oculaire	3	1,59
Prurit oculaire	10	1,59
Douleur abdominale	9	5,29
Eternuement	9	4,76
Asthénie physique	7	3,70
Anosmie	4	2,12
Anorexie	4	2,12
Agueusie	1	0,53
Frisson	1	0,53

## Discussion

La COVID-19 représente 2,59% des admissions chez les enfants au CTEPI COVID-19 de Donka pendant notre période d´étude. Un survol des publications a révélé que les enfants atteints de la COVID-19 représentaient de 1,2 à 5% des cas [9, 10] ce que corrobore nos résultats. Toutes les tranches d´âge étaient touchées avec une prédominance dans la tranche d´âge de 0 à 4 ans (38,62%) parmi cette tranche nous avons dénombré 4 nouveaux nés (dont 2 naissances au CTEPI de Donka et 2 admis avec leurs mères), ils étaient âgés respectivement de 7 et 15 jours et allaités par leurs mères dont les PCR étaient positives au COVID-19. Malgré un délai de contact de 2 semaines et plus avec leurs mères 3 PCR consécutives (J_0_, J_7_, J_14_) effectuées chez ces nouveaux nés se sont révélées négatives.

À ce jour, il n´y a pas eu de preuve que le virus peut se transmettre de la mère au bébé durant la grossesse (transmission verticale); et cela avait été observé par certains auteurs à savoir que la présence du virus n´a pas été détectée dans le liquide amniotique, le placenta ou le lait maternel des femmes enceintes infectées [11]. Cependant, la possibilité d´une transmission verticale demeure activement à l´étude puisque de récents rapports de cas en Chine ont fait état de cinq nouveau-nés positifs à la COVID-19 à 16 heures, 36 heures et deux jours de vie [6, 7]. Le sexe féminin était prédominant dans notre série, ce constat est diffèrent des résultats d´études COVID-19 chez des adultes. Il existait une nette prédominance masculine, dans les études de Wu (63,7%), Guan (58,1%) et Zhou (62%) [12-14]. Cette différence est éventuellement expliquée par la fréquence plus élevée de facteurs de risques de sévérité de la maladie dans la population masculine adulte.

La prédominance féminine dans notre étude pourrait s´expliquer par la nette supériorité de ce sexe dans la population guinéenne en générale et pédiatrique en particulier [15]. La majorité des enfants admis au CTPI de Donka résidaient dans les communes de Ratoma et de Matoto en effet, ces deux communes sont les plus vastes, plus peuplés de la ville de Conakry et avec une forte densité humaine rendant difficile le respect des mesures de prévention. Les proportions élevées d´élèves pourrait s´expliquer par le fait que ces enfants avaient un âge propice à la scolarisation ce qui suppose des rencontres de groupes (garderies, jardins, écoles, espace de loisirs) aidant à la transmission de COVID-19. Mais en réalité il faut tenir compte de la possibilité d´une transmission intra familiale du fait d´un confinement qui avait été observé sur l´ensemble du territoire avec fermeture des écoles pour une longue période (plus de 4 mois), favorisant ainsi des contacts fréquents et durables (entre adultes/enfants) dans des foyers à grande promiscuité.

Les proportions élevées des fonctionnaires et des marchandes parmi les personnes contacts des enfants de notre série expliqueraient le risque de transmission du COVID-19 que courraient ces enfants du fait que leurs proches avaient des risques élevés en fréquentant dans l´exercice de leur profession des populations (marché, transports en commun, collègues de service) ou le respect des mesures de prévention (confinement, distanciation, hygiène des mains) étaient faibles et souvent mal observées. La contamination des enfants de notre série était essentiellement d´origine intrafamiliale. Les proches (mères et/ou pères) représentaient la majorité des personnes contactes. En effet, d´après la recherche des contacts dans différents pays, l´exposition en milieu familial représentait le principal facteur de risque de contagion au COVID-19 chez les enfants selon un récent rapport aux Etats-Unis [16]. Près de 90% des enfants de notre étude avaient été exposés à la COVID-19 à la maison. Certains antécédents pathologiques des enfants de notre série avaient été décrits par des auteurs comme facteurs de risque à l´aggravation clinique des cas de COVID-19 et significativement associés á un taux de mortalité élevé [17]. Plus de la moitié de notre échantillon était asymptomatiques; ces formes semblent plus fréquentes chez l´enfant, estimées à environ 30% des cas [18].

L´impact épidémiologique de ces formes asymptomatiques n´est pas encore clair, mais plusieurs cas de contaminations durant la période d´incubation ou à partir de patients asymptomatiques ont été rapportés [18]. Une étude de Camilla Rothe au début de la crise dans l´espace de l´Union Européenne démontrait á suffisance et de façon significative que des personnes diagnostiquées positives au COVID-19 et ne présentant aucun symptôme peuvent transmettre la maladie [19]. On ne sait pas pourquoi les enfants atteints de la COVID-19 souffrent d´une maladie moins grave que les adultes. Le risque de mortalité plus élevé chez les adultes est lié au syndrome de détresse respiratoire aigu et à la défaillance multi viscérale. L´augmentation des cytokines pro-inflammatoires peut entraîner une réponse inflammatoire massive chez les adultes, qui serait atténuée pendant l´enfance [20].

La totalité des cas confirmés chez les enfants représentait moins de 2% des cas hospitalisés au CTPI de Donka. La Nouvelle-Zélande a signalé 9,6% de cas dans le groupe des 0 à 19 ans [21]. Les enfants de 19 ans et moins représentaient 4,7% des cas confirmés au Canada. Cette différence de proportions de cas pourrait être liée à la taille des échantillons étudiés. Les principaux symptômes étaient la fièvre, le nez qui coule, les céphalées, la toux, les douleurs abdominales, l´éternuement, la diarrhée, l´asthénie physique. Les enfants sont atteints d´une forme bénigne, mais il existe tout de même des cas de maladies graves [22, 23]. Nous n´avons pas rencontré de cas graves dans notre étude. La proportion des manifestations initiales diffère légèrement selon les rapports, mais environ 50% des enfants présentent une toux sèche et certains font de la fièvre (de 40 à 50%), des maux de gorge (25%), un essoufflement (13%) ou de la diarrhée (13%) ou éprouvent des malaises ou de la fatigue. Les *Centers for Disease Control and Prevention (CDC)* des États-Unis ont souligné que 745 (5,7%) des 2 572 enfants atteints de la COVID-19 ont été hospitalisés, dont 15 (0,58%) aux soins intensifs [16].

## Conclusion

Cette étude a permis de constater que la proportion de cas pédiatriques de COVID-19 est faible. L´infection pédiatrique à COVID-19 est souvent asymptomatique, bénigne et d´évolution favorable, sa transmission est quasiment intra familiale. La symptomatologie est dominée par la fièvre, la rhinorrhée, les céphalées, la toux, les douleurs abdominales, l´éternuement, la diarrhée, asthénie physique. Les transmissions verticales sont encore mal élucidées, des études à grande échelle aideraient à améliorer la compréhension de ce mécanisme et à analyser les facteurs de risque.

### Etat des connaissances sur le sujet

- De nos jours la COVID-19 demeure mondialement un problème de santé publique marqué par son impact sur la morbi-mortalité et ses conséquences économico-sociales à travers le monde;
- La COVID-19 est une maladie virale très contagieuse, dont le pronostic est grave pour les personnes du 3^e^ âge, les cas pédiatriques sont rares et plus souvent bénins, la transmission verticale est décrite.

### Contribution de notre étude à la connaissance

- la transmission verticale materno-fœtale reste une question essentielle car nous n´avons pas rencontré de cas chez les nouveau-nés, cette question mérite d´être approfondie;
- Les formes graves sont très rares chez les enfants. La mortalité des enfants par la COVID-19 est souvent faible voire quasi nulle comme dans notre série.
